# Moss occurrences in Salair-Kuznetsk Region (Altai-Sayan mountain country)

**DOI:** 10.3897/BDJ.9.e72889

**Published:** 2021-09-15

**Authors:** Olga Pisarenko

**Affiliations:** 1 Central Siberian Botanical Garden, SB RAS, Novosibirsk, Russia Central Siberian Botanical Garden, SB RAS Novosibirsk Russia

**Keywords:** dataset, specimen, moss occurrences, Bryophyta, field study, herbarium data, Altai-Sayan mountain country, bryoflora

## Abstract

**Background:**

In the flora of large regions, mosses comprise about a quarter of the total diversity of higher plants. However, now mosses are the least studied group of higher plants. Data on moss species distribution are fragmentary, especially in Russia with its vast expanse and low density of botanists.

The author for many years has been studying the bryoflora of various areas of the Salair-Kuznetsk Region. In addition to the herbarium collection, the author's bryological relevés were organised as a Database. It stores all the assembled information for the years about the locations of the species, including when the specimens were not placed in the herbarium.

The article describes three datasets that were arranged from the author's databases for three geomorphological units in the northwest of Altai-Sayan mountain country (South Siberia). Together, these three units are combined into the Salair-Kuznetsk Region. The datasets are:

1. Moss occurrences in the Kuznetsk upland.

The dataset consists of 3940 occurrence records and includes both preserved specimens (1135) and ‘human observations’ of the author (2805). The material was collected mainly from 1992-2011; some samples collected by A. N. Vasiliev in 1970-1971 (165) were also taken into account. A total of 312 moss taxa belonging to 135 genera and 41 families are reported for the region.

2. Moss occurrences in Salair Ridge.

The dataset consists of 2442 occurrence records and includes both preserved specimens (553) and ‘human observations’ of the author (1889). The material was collected mainly from 1992-1996; a total of 231 moss taxa belonging to 119 genera and 35 families are reported for the region.

3. Moss occurrences in Kuznetsk Depression.

The dataset consists of 1690 occurrence records and includes both preserved specimens (281) and ‘human observations’ of the author (1409). The material was collected mainly from 2007-2014; a total of 155 moss taxa belonging to 85 genera and 30 families are reported for the region.

All the records are geo-linked. The uncertainty of coordinates in metres varies from 500.0-10000.0 m for the earliest records that are geo-linked by topo-map, to 10.0-100.0 m for records after 2003 that are geo-linked by GPS.

**New information:**

The article summarises the results of the author's long-term bryological investigations in the Salair-Kuznetsk Region (northwest of Altai-Sayan mountain country, South Siberia).

In total, 8072 occurrence records for 366 moss species from 148 genera and 41 families are published for the territory. The datasets contribute to filling gaps in the moss species distribution and ecology.

## Introduction

The Altai-Sayan mountain country is one of the most diverse and most interesting territories in Siberia from a botanical point of view. Since the beginning of the 18^th^ century, since the first Siberian expeditions of the Russian Academy of Sciences, this territory has remained in the focus of attention of botanists in various aspects. The current state of bryological knowledge for the territory was summarised recently (Pisarenko 2020). The author's works for many years have been concentrated in the north-western, poorly studied part of the territory – on Salair Ridge, Kuznetsk Alatau, Mountane Shoria, in the Kuznetsk Depression (Pisarenko 1999, Pisarenko 2004, Pisarenko 2014). In the geomorphological and geographical literature, these territories are considered the Salair-Kuznetsk Region. All the accumulated material is presented in three regional datasets (Pisarenko 2021a, Pisarenko 2021b, Pisarenko 2021c) and is summarised by this paper.

## Project description

### Title

Mosses of Russia: phylogeny, taxonomy and biogeography

### Personnel

Olga Pisarenko

### Funding

The work is a part of the collective project of Russian bryologists to study the bryoflora of Russia. It matches the state task 'Vegetation of North Asia: diversity, ecological and geographical patterns of formation, functioning of populations' (AAAAA-A21-121011290026-9). Preserved samples are in NSK (USU_440537). Digitisation of data was supported by the Russian Science Foundation № 18-14-00121.

## Sampling methods

### Study extent

**Figure 1.**
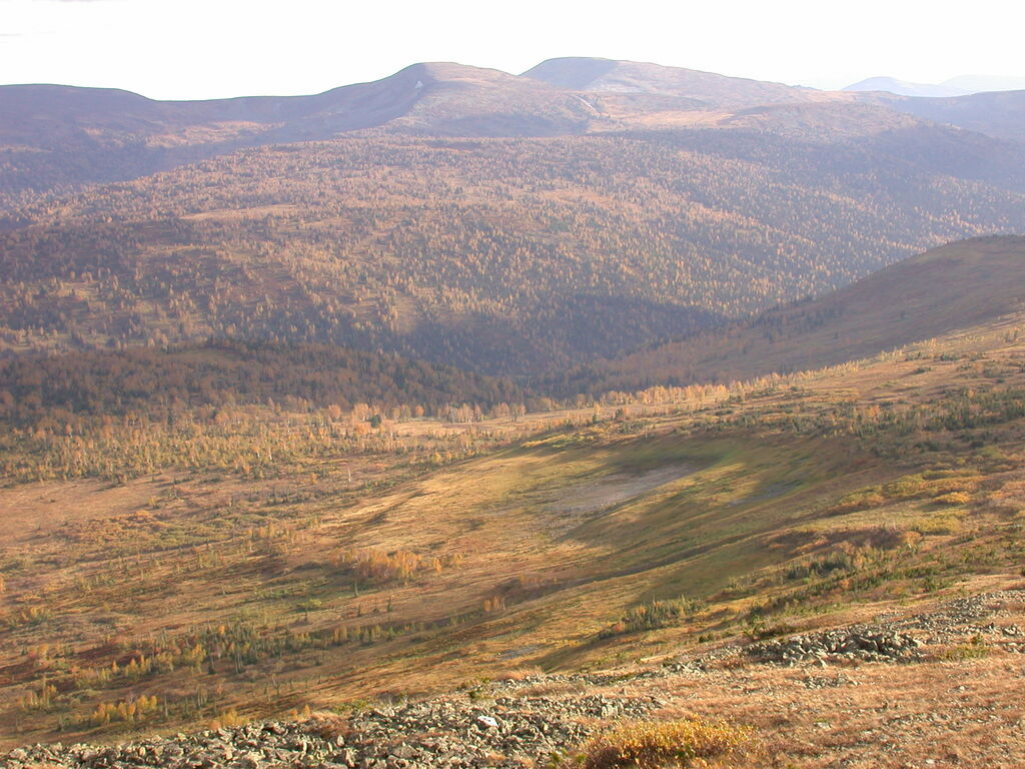
Kuznetsk Alatau, a typical picture: forest belt from mountain taiga (*Abiessibirica* dominates) on slopes, flat naked tops covered by tundra and stone fields. Photo by Olga Pisarenko.

**Figure 2.**
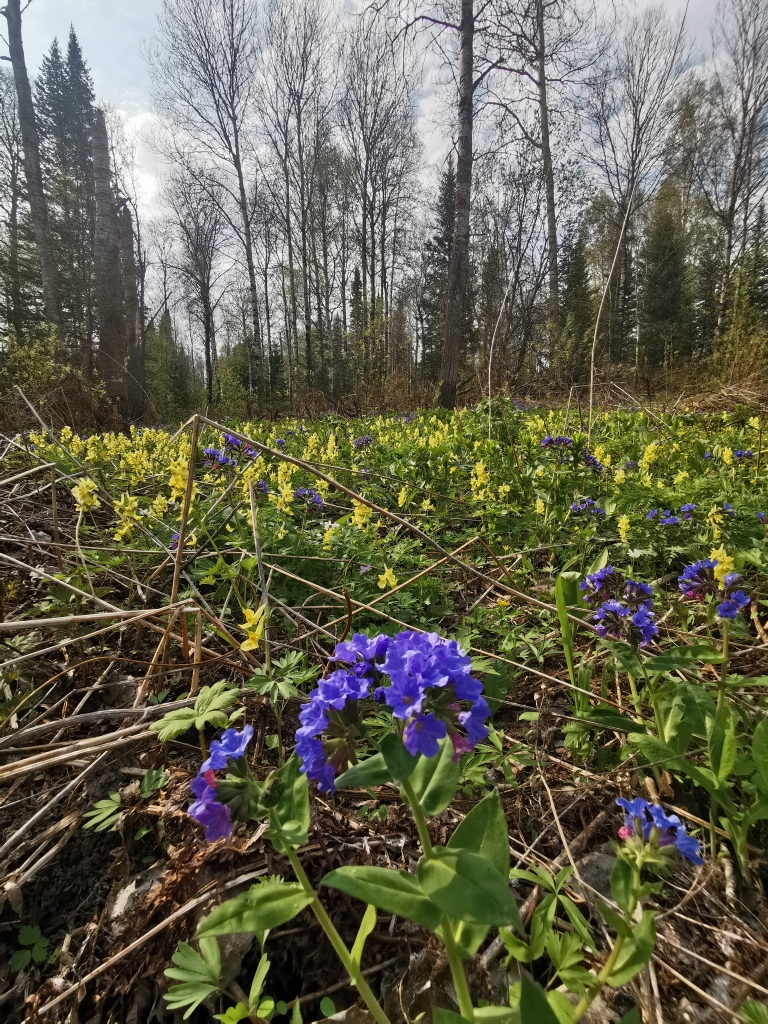
Early spring in chernevaia taiga on Salair Ridge: alternation of groups of *Populustremula* and *Abiessibirica* with herbaceous communities. Photo by Olga Pisarenko.

**Figure 3.**
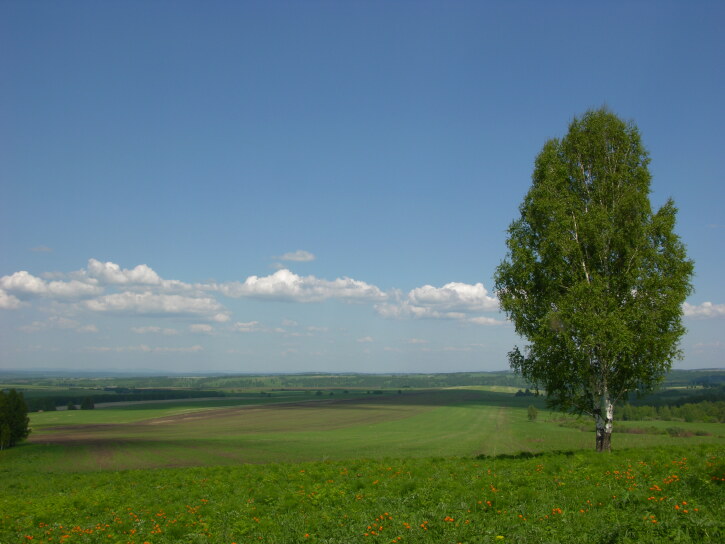
Kuznetsk Depression: forest steppe landscape now is anthropogenically strongly transformed. Photo by Olga Pisarenko.

The data paper is based on three datasets:

1. The 'mountainous' dataset (Pisarenko 2021b) presents the materials of the Mountane Shoria and the Kuznetsk Alatau (Fig. 1); it consists of 3940 occurrence records. The altitudes of the localities vary from about 200 to 1800 m. The first is the altitude along the river banks on the periphery of the mountain areas, the last is near the top of the Kanim Mt., the highest mountain of the Kuznetsky Alatau Nature Reserve. The main surveyed habitat types are: forests (mountain taiga) with predominance of *Abiessibirica*, *Pinussibirica*, *Piceaobovata*; humid and wet birch crooked forests, open mires, high-herbaceous communities, mossy thickets of *Betularotundifolia*, mountain tundra, rock outcrops and rock fields and creek banks.

2. The 'low-mountain' dataset (Pisarenko 2021a) consists of 2442 occurrence records from Salair Ridge (Fig. 2). The altitudes of the localities vary from about 140 m (river banks) to 600 m (around the highest point of the Ridge, Kivda Mt., 621 m a.s.l.). The main surveyed habitat types are: chernevaia taiga (*Abiessibirica* + *Populustremula* + high-herbaceous communities), grassy forests of *Betula*, grassy forests of *Pinussylvestris*, boggy forests of *Betula*, boggy forests of *Piceaobovata*, open sedge-moss mires, petrophytic steppes and rock outcrops.

3. The 'intermountain basins' dataset (Pisarenko 2021c) provides information on moss distribution and ecology in the Kuznetsk Depression (Fig. 3). It consists of 1690 occurrence records. The altitudes of the territory mainly are 110-330 m a.s.l., only a few localities on the sides of the basin and on the low Karakan Ridge exceed these values. The main habitat types are grassy birch and aspen forests from dry to boggy, *Salix* thickets in floodplains, mires and rock outcrops in river valleys.

### Sampling description

At the sampling stage, the task was to fully cover all types of potential moss habitats – both widespread and rare. On a series of key sites, work was carried out in a stationary or semi-stationary mode; other territories were surveyed by the route method.

The sites for the survey were planned after analysing the literature, satellite images and topographic maps. Both background and rare types of plant communities of the territory were taken into account. During field explorations, the first step in most cases was the geobotanical relevé that is important for linking bryological data with plant communities. Rock outcrops, brook banks, pass sides and so on were the objects of special attention.

On the examined plots, moss samples were picked on each type of substrate; standard methods of moss collecting were used.

After 2003, localities were georeferenced using 12-channel GPS Garmin. For earlier records, georeferencing was performed using paper maps with a scale 1:50000 - 1:500000 and maps and satellite images available by Google Earth Pro (2021) and SASGIS (2021).

All collected moss specimens were checked under the microscope and registered in the Database. Only some of the checked specimens were stored in the herbarium; preference was given to rare species, while for common species, it was down to records by ‘human observations’.

### Quality control

The data were collected and identified by researchers from the Central Siberian Botanical Garden of the Siberian Branch of the Russian Academy of Sciences.

Keys of 'Moss flora of the Middle European Russia' (Ignatov and Ignatova 2003, Ignatov and Ignatova 2004), 'Bryophyte Flora of North America' (Flora of North America Editorial Committee 2007, Flora of North America Editorial Committee 2014) and particular taxonomic articles 'Moss flora of Russia', available on arctoa.ru (Ignatov 2021) and others, were used for species identification.

Experts on different families from the Faculty of Biology of Lomonosov Moscow State University and the Tsitsin Main Botanical Garden of the Russian Academy of Sciences confirmed identifications of taxonomically difficult groups. Preserved specimens are in NSK; for the most interesting specimens, duplicates are in LE, MW and MHA.

### Step description

The species names given were determined according to the Check-list of mosses of East Europe and North Asia (Ignatov et al. 2006). Bryological survey data are stored in two personal databases. Geobotanical and bryological relevés are digitised into the IBIS (Zverev 2007). The selected samples are issued in NSK; herbarium label data are standardised into the spreadsheet. In addition to the authors’ collections, some specimens collected on the studied territory by A. N. Vasiliev, N. N. Lashchinsky and others also were included. Herbarium labels are partly available in the online database of the Moss flora of Russia (Ivanov et al. 2017).

In spring 2021, all the data were revised. Missing geo-links were added. Some recent nomenclature changes have been taken into account. Species names were matched with 'GBIF backbone' by 'Species Matching Tool' (https://www.gbif.org/tools/species-lookup). Contents of the fields were aligned with Darwin Core (Wieczorek et al. 2012). According to the territorial basis, three datasets were organised from the material. The datasets were published by GBIF (Pisarenko 2021a, Pisarenko 2021b, Pisarenko 2021c).

## Geographic coverage

### Description

**Figure 4.**
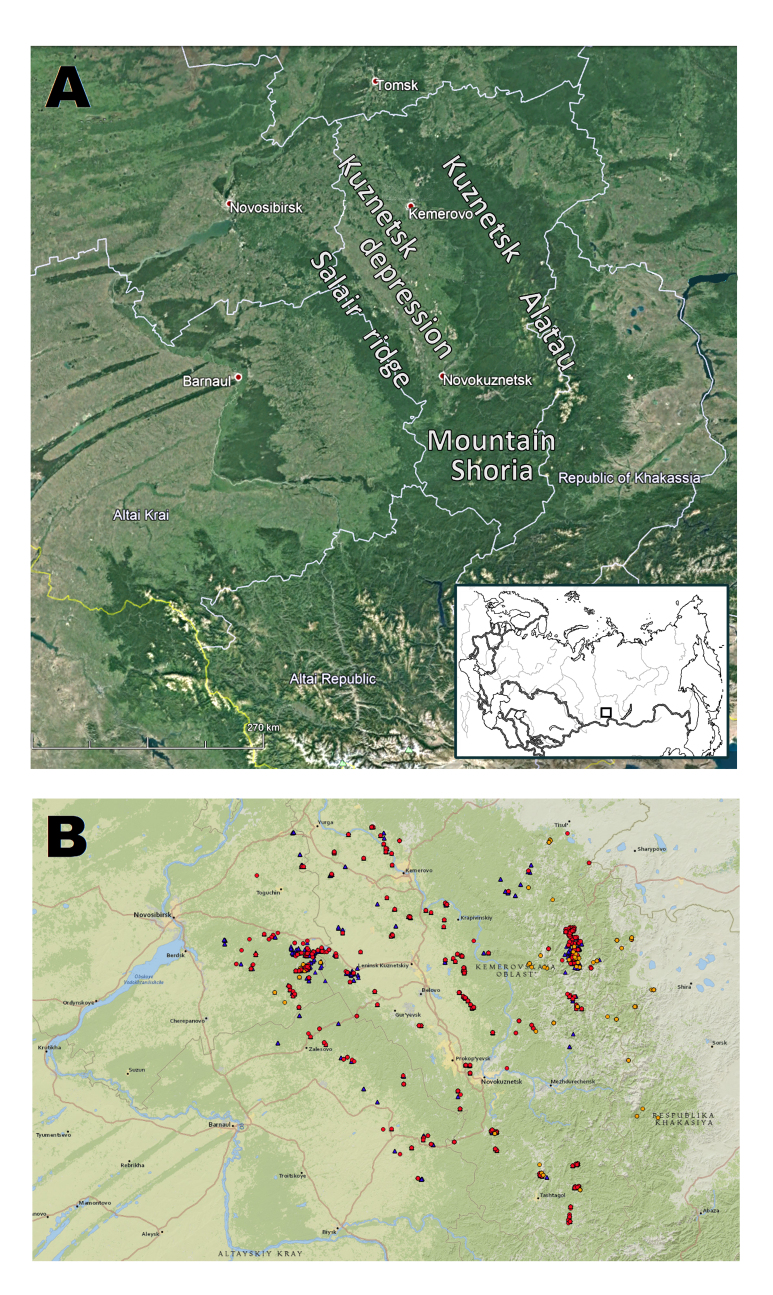
Geographic coverage: **A** Study areas in the south of Siberia; the inset shows the location in Russia; **B** Surveyed localities. Dots are preserved specimens (NSK), red dots are samples collected by the author, orange dots are samples collected by other persons; blue triangles are “human observation” of the author (usually for common species). Coordinates: 52°3'18'' and 55°50'13' Latitude; 81°6'46'' and 90°39'43' Longitude.

All the material was collected in the Asian part of Russia (Fig. 4): Kemerovo and Novosibirsk Region, Altai Territory, the Republic of Khakassia.

In the botanical and geographical zoning, the surveyed territory is considered as the Salair-Kuznetsk Province of Altai-Sayan macroprovince (Shumilova 1962). The allocation corresponds to geology. In the geomorphological literature, the Salair-Kuznetsk Region is distinguished (Olyunin 1975). That is described as a Late Mesozoic-Cenozoic gentle dome-shaped uplift, collapsed in the centre and known as the Kuznetsk Depression (Olyunin 1975). The rest are arch-block erosion-denudation mountains; in the southwest, they are low and weakly dissected (Salair Ridge); in the northeast, they are medium-high and sharply dissected, in places with glacial and char morphosculpture (the Kuznetsk Alatau and the Mountane Shoria).

The boundary between the Kuznetsk Alatau and the Mountane Shoria is drawn along the Tom River. Kuznetsk Alatau stretches northwards from the Tom River. Altitudes range from 300- 500 m a.s.l. in the northern part to 2000 m a.s.l. in the south. Elevation intervals from the level of the valleys to the highest peaks are about 600-700 m a.s.l. Flat watersheds and slightly inclined surfaces are covered with Quaternary sediments. The most widespread rocks are gabbros, granodiorites, granites, gneisses, serpentinites, clayish and crystalline slates. Limestone outcrops are rare and revealed in valleys of only some rivers. Mountane Shoria is situated southwards from the Tom River, and is characterised by smooth relief with elevation intervals between valleys and ridges being 200-300 m a.s.l. Most peaks are lower than 1200 m a.s.l. and do not exceed the tree line. The mean annual precipitation in Kuznetsk Alatau and Mountane Shoria is several times higher than in neighbouring regions and varies from 900-1000 up to 3000-3500 mm according to different data and different parts of the area. An average annual temperature is about 0°C. Winter snow depth varies from 20-40 cm on flat mountain tops to 170-190 cm in the subalpine belt. Snow spots may persist for the whole summertime on northern slopes near ridge tops. In the vegetation of the Kuznetsk upland, there are three mountain belts: a forest belt, a tall-herbaceous (subalpine) belt and a tundra belt. Mountane Shoria is a warmer region and the prevailing type of primeval vegetation here is chernevaia taiga (parcels of *Abiessibirica* and *Populustremula* with high-herbaceous spots), the most thermophilic and humid type of forest in South Siberia (Polikarpov et al. 1986). In Kuznetskiy Alatau, chernevaia taiga occurs also at the lower elevations. Forests of *Abiessibirica* with a rich herbs layer occupy slopes of all expositions from river valleys up to 1100 m a.s.l. At 1100-1250 m a.s.l., high-herbaceous communities ('subalpinotype meadows') prevail. High-mountain tundra occurs on flat tops of the upper surface of peneplainisation. Due to smooth forms of relief and the huge amount of precipitation, mires are widespread and diverse.

Salair Ridge is a low peneplain, indistinctly expressed in relief. Its average altitudes are ca. 400 m a.s.l. The ridge surface is covered with a thick layer of Quaternary sediments; rock outcrops occur mainly in river valleys. Annual precipitation is about 1000 mm on the western slope, declining to 400 mm in the rain shadow on the eastern one. The Ridge is totally within the forest belt. The largest areas are occupied by chernevaia taiga. On the steep and drier eastern macroslopes of the Ridge, mesophilic grassy pine and birch forests prevail. Petrophytic steppe occurs on steep and sunny faces amongst the forest. Mires are rare and small.

Kuznetsk Depression is an elevated hilly plain; altitudes are 110-300 m a.s.l. The main part of the Depression is within the forest-steppe zone; zonal vegetation is composed of *Betula*-forests in combination with steppe meadows and meadow steppes. Mires are very rare, occurring only in river valleys. Rock outcrops in the Kuznetsk Depression are also associated with river valleys. The plant cover of the Depression is strongly transformed by agriculture and mining activities: now it is very difficult to find fragments of native vegetation.

A detailed description of the nature of the region and references have been recently published by the author (Pisarenko 2014).

### Coordinates

52.05 and 55.84 Latitude; 81.11 and 90.66 Longitude.

## Taxonomic coverage

### Description

A total of 366 moss species from 148 genera and 41 families according to 'GBIF backbone', by 'Species Matching Tool' (https://www.gbif.org/tools/species-lookup).

It should be borne in mind that the understanding of taxa in the 'GBIF backbone' differs significantly both from the Checklist (Ignatov et al. 2006) and from the latest interpretation of species, families and genera of mosses (Hodgetts et al. 2020, Ignatov 2021). Therefore, these numbers should be considered as a rough estimation of moss diversity of the region.

In total, 8072 occurrence records are published for the Salair-Kuznetsk Region in the three datasets. All mosses are identified to species.

Many species are known in the studied territory from single localities, although the number of records may not be single in case if, in this single locality, species occupy different microhabitats. The first hundred of the rarest species in the studied territory are *Aloinabrevirostris*, *Anacamptodonlatidens*, *Brachytheciumcomplanatum*, *Bryumschleicheri*, *Bryumuliginosum*, *Bryumweigelii*, *Campylophyllumhalleri*, *Cinclidiumstygium*, *Cinclidotusriparius*, *Cynodontiumtenellum*, *Cyrtomniumhymenophylloides*, *Dichelymafalcatum*, *Dicranodontiumdenudatum*, *Dicranumacutifolium*, *Dicranumbrevifolium*, *Didymodonferrugineus*, *Ditrichumheteromallum*, *Ditrichumpusillum*, *Drepaniumrecurvatum*, *Drepanocladussendtneri*, *Encalyptatrachymitria*, *Fabroniaciliaris*, *Fontinalishypnoides*, *Grimmiaalpestris*, *Grimmiaanodon*, *Grimmiaanomala*, *Grimmiacaespiticia*, *Grimmiafunalis*, *Grimmialaevigata*, *Grimmiateretinervis*, *Grimmiatergestina*, *Grimmiaunicolor*, *Hamatocaulisvernicosus*, *Haplocladiumangustifolium*, *Herzogiellastriatella*, *Herzogiellaturfacea*, *Isopterygiopsisalpicola*, *Isopterygiopsiscatagonioides* (as *I.muellerianain* 'GBIF backbone'), *Iwatsukiellaleucotricha*, *Kiaeriaglacialis*, *Lescuraeasecunda*, *Meesiauliginosa*, *Mniumspinulosum*, *Mniumthomsonii*, *Myriniapulvinata*, *Myurellatenerrima*, *Neckerabesseri*, *Oligotrichumhercynicum*, *Orthotheciumintricatum*, *Orthotrichumalpestre*, *Palustriellacommutata*, *Palustriellafalcata*, *Paraleucobryumenerve*, *Physcomitriumpyriforme*, *Physcomitriumpyriforme*, *Plagiotheciumlatebricola*, *Platydictyajungermannioides*, *Podperaeakrylovii*, *Pohlialongicollis*, *Pohliamelanodon*, *Pohliaproligera*, *Polytrichumpallidisetum*, *Pseudephemerumnitidum*, *Pseudocalliergonlycopodioides*, *Pseudocalliergontrifarium*, *Pterygoneurumovatum*, *Pterygoneurumsubsessile*, *Racomitriumlanuginosum*, *Rhabdoweisiacrispata*, *Rhizomniumandrewsianum*, *Rhodobryumontariense*, *Rhynchostegiumarcticum*, *Rhynchostegiumrotundifolium*, *Schistidiumcrenatum*, *Schistidiumobscurum*, *Schistidiumplatyphyllum*, *Schistidiumsibiricum*, *Schistidiumsinensiapocarpum*, *Schistidiumtenuinerve*, *Scorpidiumcossonii*, *Scorpidiumscorpioides*, *Seligeriabrevifolia*, *Seligeriadonniana*, *Seligeriatristichoides*, *Sphagnumcompactum*, *Sphagnumcontortum*, *Sphagnummajus*, *Sphagnumobtusum*, *Sphagnumpalustre*, *Sphagnumpalustre*, *Sphagnumteres*, *Tetraplodonmnioides*, *Timmiabavarica*, *Tortellaalpicola*, *Tortulaacaulon*, *Tortulamucronifolia*, *Tortulamuralis*, *Trachycystisussuriensis*, *Trichostomumcrispulum* and *Zygodonsibiricus*. In the three datasets, the share of these 100 species is 2.6% of all occurrence records.

The largest number of records are referred to the following species: *Pleuroziumschreberi* (212 records), *Sanioniauncinata* (211), *Plagiomniumcuspidatum* (206), *Sciuro*-*hypnum reflexum* (199), *Plagiotheciumdenticulatum* (155), *Plagiomniumellipticum* (151), *Amblystegiumserpens* (145), *Brachytheciumsalebrosum* (147), *Pylaisiapolyantha* (129), *Callicladiumhaldanianum* (117), *Rhodobryumroseum* (115), *Aulacomniumpalustre* (113), *Ceratodonpurpureus* (109), *Climaciumdendroides* (102) and *Pohlianutans* (102). Thus, for 15 species, there are more than 100 records for each; these 15 species account for 27.4% of all occurrence records.

The richest ten families by the number of species are Dicranaceae (36 species), Amblystegiaceae (35), Grimmiaceae (32), Brachytheciaceae (27), Bryaceae (26), Sphagnaceae (24), Pottiaceae (24), Mniaceae (20), Hypnaceae (17) and Polytrichaceae (15).

### Taxa included

**Table:** 

Rank	Scientific Name	
phylum	Bryophyta	
kingdom	Plantae	
class	Sphagnopsida	
class	Andreaeopsida	
class	Polytrichopsida	
class	Tetraphidopsida	
class	Bryopsida	

## Traits coverage

### Data coverage of traits

PLEASE FILL IN TRAIT INFORMATION HERE

## Temporal coverage

### Notes

Mainly 1992-2011 for DOI: 10.15468/skqw4k; mainly 1992-1996 for DOI: 10.15468/g4cmuh; mainly 2007-2014 for DOI: 10.15468/vdxqsr.

## Collection data

### Collection name

USU_440537 Herbarium of higher plants, lichens and fungi (NS, NSK); Central Siberian Botanical Garden SB RAS

### Collection identifier

NSK

### Parent collection identifier

https://csbg-nsk.ru/unu_herbarium

### Specimen preservation method

dried аnd pressed

## Usage licence

### Usage licence

Other

### IP rights notes

This work is licensed under a Creative Commons Attribution Non Commercial (CC-BY-NC) 4.0 License.

## Data resources

### Data package title

Moss occurrences in Salair-Kuznetsk Region (Altai-Sayan mountain country)

### Number of data sets

3

### Data set 1.

#### Data set name

Moss occurrences in the Kuznetsk upland (Altai-Sayan mountain country)

#### Data format

Darwin Core Archive format.

#### Number of columns

31

#### Character set

UTF-8

#### Download URL

https://doi.org/10.15468/skqw4k

#### Data format version

Darwin Core Archive format.

#### Description

The dataset contains information on moss occurrences in the territory of Kuznetsk upland, which is situated in the northwest of Altai-Sayan mountain country, South Siberia. The dataset summarises data of the author's bryological explorations of the territory; the surveys were mainly conducted from 1992-2011. A.N. Vasiliev was working on the territory from 1970-1971; part of the collection is in NSK, the samples processed by the author being included in this dataset.

The dataset consists of 3940 occurrence records and includes both preserved specimens (1135) and the ‘human observations’ of the author (2805). The ‘human observations’ are data of bryological relevés. The relevés were made during field explorations; all collected moss specimens were checked under the microscope and registered in Data Base. Only some of the checked specimens were stored in the herbarium; preference was given to rare species, but for common species, it was down to notes. All the records are georeferenced.

A total of 312 moss taxa, belonging to 135 genera and 41 families, are reported herein to occur in the Kuznetsk upland.

The work is supported by the Russian Science Foundation № 18-14-00121

**Data set 1.** 

Column label	Column description
occurrenceID	An identifier for the record, unique within this dataset. An abbreviation in the identifier' number (kuzn_upland-xxxx).
taxonID	An identifier for the set of taxon information (data associated with the Taxon class).
kingdom	The full scientific name of the kingdom in which the taxon is classified.
phylum	The full scientific name of the phylum or division in which the taxon is classified.
class	The full scientific name of the class in which the taxon is classified.
family	The full scientific name of the family in which the taxon is classified.
genus	The full scientific name of the genus in which the taxon is classified.
scientificName	The full scientific name, with authorship and date information, if known.
taxonRank	The taxonomic rank.
acceptedNameUsage	The full name, with authorship and date information, if known, of accepted taxon.
country	Country name (Russian Federation).
countryCode	The standard code for the Russian Federation according to ISO 3166-1-alpha-2 (RU).
stateProvince	Region (‘oblast’) name. The first-level administrative division.
county	District (‘raion’) name. The secomd-level administrative division
verbatimLocality	The original textual description of the place, in Russian.
locationRemarks	Comments or notes about the Location.
verbatimElevation	Altitude above sea level.
decimalLatitude	The geographic latitude in decimal degrees of the geographic centre of the data sampling place.
decimalLongitude	The geographic longitude in decimal degrees of the geographic centre of the data sampling place.
coordinateUncertaintyInMetres	The maximum uncertainty distance in metres.
habitat	A category or description of the habitat in which the Event occurred.
fieldNotes	The text of notes taken in the field about the Event, in Russian.
year	The four-digit year in which the Event occurred, according to the Common Era Calendar.
month	The integer month in which the Event occurred.
day	The integer day of the month on which the Event occurred.
recordedBy	List of persons who collected field data.
identifiedBy	A person who assigned the Taxon to the subject.
basisOfRecord	The specific nature of the data record in standard label of one of the Darwin Core classes: PreservedSpecimen/HumanObservation.
collectionCode	The acronym from which the record for preserved sample was derived (NSK).
catalogNumber	An identifier (preferably unique) for the record within the NSK collection.
datasetName	The name identifying the dataset from which the record was derived (NSK20xxxxx).

### Data set 2.

#### Data set name

Moss occurrences in Salair Ridge (Altai-Sayan mountain country).

#### Data format

Darwin Core Archive format.

#### Number of columns

31

#### Character set

UTF-8

#### Download URL

https://doi.org/10.15468/g4cmuh

#### Description

The dataset contains information on moss occurrences in the territory of Salair Ridge, which is the northwest spoor of Altai-Sayan mountain country, South Siberia. The dataset summarises data of the author's bryological explorations of the territory. The surveys were mainly conducted from 1992-1996, with some later visits on the Ridge.

The dataset consists of 2439 occurrence records and includes both preserved specimens (553) and ‘human observations’ of the author (1886). The ‘human observations’ are data of bryological relevés. The relevés were made during field explorations; all collected moss specimens were checked under the microscope and registered in the Database. Only some of the checked specimens were stored in the herbarium; preference was given to rare species, but for common species, it was down to notes. All the records are georeferenced. A total of 231 moss taxa, belonging to 119 genera and 35 families, are reported herein to occur in Salair Ridge.

The work is under the support of Russian Science Foundation № 18-14-00121, https://rscf.ru/project/18-14-00121/.

**Data set 2.** 

Column label	Column description
occurrenceID	An identifier for the record, unique within this dataset. An abbreviation in the identifier' number (salair-xxxx).
taxonID	An identifier for the set of taxon information (data associated with the Taxon class).
kingdom	The full scientific name of the kingdom in which the taxon is classified.
phylum	The full scientific name of the phylum or division in which the taxon is classified.
class	The full scientific name of the class in which the taxon is classified.
family	The full scientific name of the family in which the taxon is classified.
genus	The full scientific name of the genus in which the taxon is classified.
scientificName	The full scientific name, with authorship and date information, if known.
taxonRank	The taxonomic rank.
acceptedNameUsage	The full name, with authorship and date information, if known, of accepted taxon.
country	Country name (Russian Federation).
countryCode	The standard code for the Russian Federation according to ISO 3166-1-alpha-2 (RU).
stateProvince	Region (‘oblast’) name. The first-level administrative division.
county	District (‘raion’) name. The secomd-level administrative division.
verbatimLocality	The original textual description of the place, in Russian.
locationRemarks	Comments or notes about the Location.
verbatimElevation	Altitude above sea level.
decimalLatitude	The geographic latitude in decimal degrees of the geographic centre of the data sampling place.
decimalLongitude	The geographic longitude in decimal degrees of the geographic centre of the data sampling place.
coordinateUncertaintyInMetres	The maximum uncertainty distance in metres.
habitat	A category or description of the habitat in which the Event occurred.
fieldNotes	The text of notes taken in the field about the Event, in Russian.
year	The four-digit year in which the Event occurred, according to the Common Era Calendar.
month	The integer month in which the Event occurred.
day	The integer day of the month on which the Event occurred.
recordedBy	List of persons who collected field data.
identifiedBy	A person who assigned the Taxon to the subject.
basisOfRecord	The specific nature of the data record in standard label of one of the Darwin Core classes: PreservedSpecimen/HumanObservation.
collectionCode	The acronym from which the record for preserved sample was derived (NSK).
catalogNumber	An identifier (preferably unique) for the record within the NSK collection.
datasetName	The name identifying the data set from which the record was derived (NSK20xxxxx).

### Data set 3.

#### Data set name

Moss occurrences in Kuznetsk Depression (Altai-Sayan mountain country)

#### Data format

Darwin Core Archive format.

#### Number of columns

31

#### Character set

UTF-8

#### Download URL

https://doi.org/10.15468/vdxqsr

#### Description

The dataset contains information on moss occurrences in the territory of Kuznetsk Depression. The Depression is located in the north-west of Altai-Sayan mountain country, South Siberia; from the east, it is limited by the Kuznetsk Alatau, from the south - by the Mountain Shoria, from the west - by Salair Ridge. The dataset summarises data of the author's bryological explorations of the territory. The surveys were mainly conducted from 2007-2014. The dataset consists of 1690 occurrence records and includes both preserved specimens (281) and ‘human observations’ of the author (1409). The ‘human observations’ are data of bryological relevés. The relevés were made during field explorations; all collected moss specimens were checked under the microscope and registered in Data Base. Only some of the checked specimens were stored in the herbarium; preference was given to rare species, but for common species, it was down to notes. All the records are georeferenced. A total of 155 moss taxa belonging to 85 genera and 30 families are reported herein to occur in Salair Ridge. Preserved samples are in NSK (USU_440537). The work is supported by the Russian Science Foundation № 18-14-00121.

**Data set 3.** 

Column label	Column description
occurrenceID	An identifier for the record, unique within this dataset. An abbreviation in the identifier' number (kuzn_depres-xxxx).
taxonID	An identifier for the set of taxon information (data associated with the Taxon class).
kingdom	The full scientific name of the kingdom in which the taxon is classified.
phylum	The full scientific name of the phylum or division in which the taxon is classified.
class	The full scientific name of the class in which the taxon is classified.
family	The full scientific name of the family in which the taxon is classified.
genus	The full scientific name of the genus in which the taxon is classified.
scientificName	The full scientific name, with authorship and date information if known.
taxonRank	The taxonomic rank.
acceptedNameUsage	The full name, with authorship and date information, if known, of accepted taxon.
country	Country name (Russian Federation).
countryCode	The standard code for the Russian Federation according to ISO 3166-1-alpha-2 (RU).
stateProvince	Region (‘oblast’) name. The first-level administrative division.
county	District (‘raion’) name. The secomd-level administrative division.
verbatimLocality	The original textual description of the place, in Russian.
locationRemarks	Comments or notes about the Location.
verbatimElevation	Altitude above sea level.
decimalLatitude	The geographic latitude in decimal degrees of the geographic centre of the data sampling place.
decimalLongitude	The geographic longitude in decimal degrees of the geographic centre of the data sampling place.
coordinateUncertaintyInMetres	The maximum uncertainty distance in metres.
habitat	A category or description of the habitat in which the Event occurred.
fieldNotes	The text of notes taken in the field about the Event, in Russian.
year	The four-digit year in which the Event occurred, according to the Common Era Calendar.
month	The integer month in which the Event occurred.
day	The integer day of the month on which the Event occurred.
recordedBy	List of persons who collected field data.
identifiedBy	A person who assigned the Taxon to the subject.
basisOfRecord	The specific nature of the data record in standard label of one of the Darwin Core classes: PreservedSpecimen/HumanObservation.
collectionCode	The acronym from which the record for preserved sample was derived (NSK).
catalogNumber	An identifier (preferably unique) for the record within the NSK collection.
datasetName	The name identifying the data set from which the record was derived (NSK20xxxxx).
